# Pixel-Level Fatigue Crack Segmentation in Large-Scale Images of Steel Structures Using an Encoder–Decoder Network

**DOI:** 10.3390/s21124135

**Published:** 2021-06-16

**Authors:** Chuanzhi Dong, Liangding Li, Jin Yan, Zhiming Zhang, Hong Pan, Fikret Necati Catbas

**Affiliations:** 1 Department of Civil, Environmental, and Construction Engineering, University of Central Florida, Orlando, FL 32816, USA; catbas@ucf.edu; 2 Department of Computer Science, University of Central Florida, Orlando, FL 32816, USA; leoriohope@knights.ucf.edu; 3 Palo Alto Research Center, Palo Alto, CA 94304, USA; jyan@parc.com; 4 School for Engineering of Matter, Transport and Energy, Arizona State University, Tempe, AZ 85281, USA; zzhan506@asu.edu; 5 Department of Civil and Environmental Engineering, North Dakota State University, Fargo, ND 58105, USA; hong.pan@ndsu.edu

**Keywords:** fatigue crack, steel structures, computer vision, semantic segmentation, deep learning

## Abstract

Fatigue cracks are critical types of damage in steel structures due to repeated loads and distortion effects. Fatigue crack growth may lead to further structural failure and even induce collapse. Efficient and timely fatigue crack detection and segmentation can support condition assessment, asset maintenance, and management of existing structures and prevent the early permit post and improve life cycles. In current research and engineering practices, visual inspection is the most widely implemented approach for fatigue crack inspection. However, the inspection accuracy of this method highly relies on the subjective judgment of the inspectors. Furthermore, it needs large amounts of cost, time, and labor force. Non-destructive testing methods can provide accurate detection results, but the cost is very high. To overcome the limitations of current fatigue crack detection methods, this study presents a pixel-level fatigue crack segmentation framework for large-scale images with complicated backgrounds taken from steel structures by using an encoder-decoder network, which is modified from the U-net structure. To effectively train and test the images with large resolutions such as 4928 × 3264 pixels or larger, the large images were cropped into small images for training and testing. The final segmentation results of the original images are obtained by assembling the segment results in the small images. Additionally, image post-processing including opening and closing operations were implemented to reduce the noises in the segmentation maps. The proposed method achieved an acceptable accuracy of automatic fatigue crack segmentation in terms of average intersection over union (mIOU). A comparative study with an FCN model that implements ResNet34 as backbone indicates that the proposed method using U-net could give better fatigue crack segmentation performance with fewer training epochs and simpler model structure. Furthermore, this study also provides helpful considerations and recommendations for researchers and practitioners in civil infrastructure engineering to apply image-based fatigue crack detection.

## 1. Introduction

### 1.1. Background

With the advantages of both compression and tension capacity, light self-weight, easy fabrication, simple construction, steel bridges have been widely constructed in the world over hundreds of years [1]. The high performance of steel structures makes the steel bridge types suitable for different span ranges, including short-span (up to 15 m), medium-span (up to 50 m), and long-span (up to 1000 m) scenarios. However, during the long-term operation stage of steel bridges, damages would occur gradually in the steel components due to the effects of increasing traffic loads, overloading trucks, deficiencies of welding joints and materials, stress concentration, corrosion, and the complex interactions of multiple factors [2]. The initial tiny damages will accumulate and eventually evolve to the fatigue cracks [3] in the steel components due to the repeated stress induced by daily traffic loads and the distortion-induced fatigue cracks due to secondary stresses in the steel components that comprise bridge member cross-sections [4]. The fatigue cracks would result in the loss of bridge load carrying capacity, lead to early permit post or termination of services. It is essential to detect and monitor the fatigue cracks in steel bridges in time to support the decision-making of bridge maintenance and retrofit procedures, extend the life cycles, and prevent early bridge failures.

In current research and engineering practices, visual inspection is the primary approach for the inspection of fatigue cracks. Bridge owners and bridge management departments put large amounts of effort and resources into the visual inspection task, guideline, and personnel [5]. A qualified inspector needs to go through the qualification, training, and certification of inspectors [6]. However, the accuracies of visual inspection highly rely on the inspectors’ subjective judgments. According to Campbell et al.’s recent study [6], the experience and training of inspectors, the field temperature during visual inspection, and the inspection duration were significantly correlated with the detection rate. The objectives of the visual inspection items in their study were exactly fatigue cracks in steel bridges. In addition, the amount of time, labor forces, and cost spent during the visual inspection is quite large and cannot be ignored. Non-destructive testing (NDT) methods that utilize advanced sensing technologies can greatly reduce the inspection errors and subjectivities compared to the conventional visual inspection. The most common NDT inspection methods include radiography, ultrasound, dye-penetrant and magnetic-particle testing, and acoustic emission [7]. These NDT methods can provide quantitative inspection results [8], but they require close physical access or contact detection with external excitation/impact [9]. In addition, these NDT methods are very expensive and difficult to be widely promoted to large populations of bridges. It is necessary to develop a more effective method with higher accuracy and less spending in terms of time, cost, and labor force for the detection of fatigue cracks in steel bridges.

### 1.2. Objective and Scope

With the development of advanced imaging devices and computer vision, vision-based structural monitoring and inspection have gained increasing attention in the field of civil infrastructure monitoring [10,11,12,13,14,15,16]. Computer vision techniques can be implemented into civil infrastructure applications to track the global motions/deformations of structures [17,18,19,20,21] and detect the local changes/damages [22] with the advantages of being non-contact, long-distance, low-cost, and less time-consuming and allowing automatic monitoring and inspection. In this study, the scope and objectives are: (1) to propose a computer vision-based method for fatigue crack detection of steel bridges; (2) to achieve pixel-level crack segmentation from large-scale images (larger than 4K images) collected by consumer-grade cameras; and (3) to provide helpful considerations and recommendations for researchers and engineering practitioners in the field of civil infrastructure engineering.

## 2. Related Work

There are mainly two different types of computer vision-based fatigue crack detection approaches in the literature according to the nature of fatigue cracks: (1) detection from motion and (2) detection from still images. This section will review the related work belonging to each of these two categories, respectively.

### 2.1. Fatigue Crack Detection from Motion

This type of detection method is originally from the formation of fatigue cracks. Fatigue cracks are initiated due to tiny structural deficiencies and propagate along with the repeated loads. Based on the motion around the fatigue cracks, the crack tip and path can be depicted and estimated. Delenbaugh et al. [23] tracked the full field motion of the web-gap of a steel girder by using three-dimensional (3D) digital image correlation (DIC), and based on the full-field strain and displacement maps, they obtained the visualized fatigue crack results of the web-gap. This method for fatigue crack detection highly relies on the accuracy of the measured full-field strain and displacement data. The estimated result may need to be checked to make sure the prediction is accurate [24]. Kong and Li [13] extracted the feature points around the cut slot of a steel plate on a universal test machine. These feature points were regarded as the representatives of the cut slot. Then, they applied optical flow to track the motion of the feature points on the steel plate under repeated load cycles. Based on the time histories of the feature points, they extracted the initialization and propagation of the fatigue cracks around the cut slot of the steel plate. The main advantage of the motion-based detection methods, as mentioned above for fatigue cracks, can detect the initialization and propagation of the fatigue cracks from videos with structural motion. However, this type of method highly relies on external excitations, i.e., repeated load cycles or other types of external loads to estimate the motion and then identify fatigue crack areas. Fatigue cracks are initiated and propagated by external loads, and the necessary condition of using detection methods from motion is that there is enough motion/change in the image sequences of continuous monitoring or between the current detection image and a baseline. For the existing fatigue cracks in a relatively still environment without enough external excitations, it is very difficult to estimate the motion/change around the fatigue cracks, and it is not easy to recognize the fatigue cracks based on this type of fatigue crack detection method.

### 2.2. Fatigue Crack Detection from Still Images

The second type of method is appropriate for detecting existing fatigue cracks in still images, rather than the motion videos or consecutive image sequences with structural change. It is a more straightforward way compared to the first type of methods. Technically, the second type of method does not rely on the nature of or the reason for “fatigue” to detect the fatigue cracks. By implementing the computer vision algorithms to extract the surface features, patterns, or morphologies from the images, the location and shape of the fatigue cracks can be detected. Depending on the applied processing strategies and computer vision algorithms, the crack detection methods from still images can be further divided into two types of approaches: (1) patch-level crack detection and (2) pixel-level crack detection [12].

#### 2.2.1. Patch-Level Fatigue Crack Detection

In the patch-level crack detection approaches, the basic idea is that an image classifier for cracks is trained with small image patches dataset (e.g., 64 × 64 pixels) cropped from large images (1920 × 1080 pixels) to classify whether there are cracks inside the patches. The trained image classifier is then applied to detect the crack region inside a large image by using a sliding window or dividing the large images into different patches according to the size of the predefined patch size. Finally, the crack regions in the large image can be recognized by connecting all the detected small patches. The advantages of the patch-level crack detection approaches are as follows: (1) it is easy to train a prediction model with different types of algorithms, including the conventional machine learning methods or deep learning methods; (2) the model could be trained with a small dataset; and (3) they can give the crack detection results at different levels. For example, Chen et al. [25] trained an image classifier to detect cracks on the metallic surface using the conventional machine vision method: support vector machine (SVM), local binary patterns, and Bayesian decision theory. By sliding the image classifier on the large test image, they extracted the possible crack regions. Zhang et al. [26] trained seven different machine learning models for classification including SVM, k-nearest neighbor (KNN), Naive Bayes, decision tree, random forest, an ensemble model, and neural network (NN) to classify the crack status of small image patches (32 × 32 pixels), which were cropped from the original images (577 × 314 pixels) captured in fatigue tests. They found that the decision tree gave the best results among all the machine learning models with small training dataset. By assembling all the classification results of the image patches, the crack propagation direction and length (accuracy of 0.6 mm) can be estimated. Xu et al. [10,22] trained the image classifiers with image patches of fatigue cracks by using deep learning methods such as a convolutional neural network (CNN) and restricted Boltzmann machines algorithms. With the image crop or sliding window strategy, they applied the trained image classifier to extract the fatigue crack distributions of steel structures. There are two main limitations of the patch-level crack detection approaches. The first one is that this type of approach can only give the approximate region of cracks, rather than the detailed crack shape, morphology, and distribution map at pixel level. Moreover, it is also hard to get the size of the cracks for the purpose of structural condition assessment. To overcome this limitation, Xu et al. [10] implemented edge detection inside the detected crack patches for post-processing and eventually obtained the fatigue crack at pixel level. Wang et al. [27] applied the semantic segmentation algorithms to segment the cracks at the pixel level in the image patches, which were classified as “crack” status by CNN-based classifier. The second limitation is that the size of the patch for the image classifier can affect the detection accuracy. The patch size would affect the extraction of local and global features in the images for fatigue crack detection. Too small or too large patch sizes might both affect the detection accuracy. According to Xu et al. [22], the optimal patch size for the training of image classifier of fatigue crack detection in steel structures does exists. However, it might need to take a large amount of efforts to figure out the best patch size. This limitation might be resolved by first using the region-based object detection method to detect the region of the cracks with a suitable bounding box. The size of the bounding box is defined based on the detection result. Then, the cracks inside the bounding box are segmented at pixel level by using edge detection or semantic segmentation algorithm. Currently, there is no such application in the literature to detect fatigue cracks in steel structures. However, there are examples for crack detection in concretes using the abovementioned strategy. Karaaslan et al. [28] applied the single shot multibox detector (SSD) algorithm to detect the crack regions on the surface of concrete structures and then applied a semantic segmentation algorithm to segment the cracks at pixel level.

#### 2.2.2. Pixel-Level Fatigue Crack Detection

The basic idea of the implementation of pixel-level crack detection is to conduct just one end-to-end image segmentation model to detect the fatigue cracks at the pixel level. It is not necessary to conduct an image classification procedure in the image patches with fixed sizes, or object detection to recognize cracks in bounding boxes first and then perform segmentation inside the patches. The segmentation is directly performed on the images without initial detection, making the detection procedure much more straightforward. The shape, pattern, morphology, and distribution of the fatigue crack are visualized and presented, which makes it much easier to get the size (physical or geometrical) information of fatigue cracks for structural condition assessment. A direct way of achieving pixel-level fatigue crack detection is to apply image processing methods such as edge detection in a manual way. However, this kind of method highly relies on the manually adjusted parameters of the selected filter, thresholds, etc. Furthermore, it can introduce large amounts of noises during manual image processing because the edges that are actually not fatigue cracks can be regarded as real fatigue cracks by mistake.

Semantic segmentation is a good way to segment the cracks and especially with the aid of deep learning and large prelabelled datasets, semantic segmentation can be achieved as pixel-level classification. The fatigue crack can be detected at pixel level with the deep learning-based semantic segmentation. In the literature, there are quite few studies related to fatigue crack detection directly using deep-learning-based semantic segmentation, except for the research conducted by Wang et al. [27], in which they implemented fully convolution networks (FCN) to segment the fatigue cracks from the pre-classified image patches (already classified as images patches with fatigue cracks). Currently, most of the applications using deep learning-based semantic segmentation are related to concrete/pavement crack detection. For example, Dung and Anh [29] retrained the original FCN to segment cracks from concretes at pixel level. The original FCN [30] consists of two main parts: encoder and decoder. The encoder part generally plays a role in image feature extraction and classification and the decoder part builds the semantic segmentation map by conducting a trainable upsampling procedure. The encoder and decoder are connected in three levels, and features extracted in the encoder part at different scale levels are added to the corresponding layers of the decoder part for trainable upsampling purposes. Moreover, the authors of the original FCN [30] selected a pretrained CNN for classification, i.e., VGG16 [31] as the backbone. Theoretically, the backbone for the encoder part can be replaced with other CNN architectures such as VGG19 [31], InceptionV3 [32], ResNet152 [33], etc. In Dung and Anh’s work [29], they compared the performances of VGG16, InceptionV3, and ResNet152 on an open-source dataset of concrete crack images. They found that VGG16 showed the best results for crack classification among the three architectures, and eventually they implemented VGG16 as the backbone of FCN for crack segmentation. Yang et al. [34] implemented VGG19 as the backbone of FCN to segment the cracks in concretes and pavements. Bang et al. [35] developed an FCN architecture to segment the cracks in pavements by applying ResNet152 as the backbone of the encoder part and using three deconvolutional layers of ZFNet for the upsampling in the decoder part. In addition to FCN and its modified versions, U-net [36], which was built upon FCN, has gained increasing attention in the field of local damage detection. U-net was originally developed for the binary semantic segmentation of neuronal structures in electron microscopic stacks. Liu et al. [37] implemented U-net to segment the cracks from concretes. Shi et al. [38] proposed VGG-Unet to replace the original backbone of U-net with VGG net to segment the corrosion and cracks around the bearings of a bridge. Zhang et al. [39] investigated U-net architecture with different depths and found that with deeper architectures, the model can give better accuracy for concrete crack segmentation under complex conditions. Cui et al. [40] implemented the Att-Unet to segment the crack in the pavement and the results showed that Att-Unet increased the intersection over union (IOU), which is a key indicator to evaluate the performance of the segmentation model, from 70.44% to 73.65%. Aslam et al. [41] implemented U-net to segment the scratches on the surface of the metal.

Mei and Gul [42] developed a conditional Wasserstein generative adversarial network (cWGAN) by integrating the DenseNet121 as the encoder, a set of deconvolution layers as the decoder, and a discriminator to train a model for pavement crack detection. A generative adversarial network (GAN) usually includes a generator and a discriminator. In Mei and Gul’s work, the generator is constituted by the DenseNet121 [43] backbone and the deconvolution layers, which is actually a modified version of FCN. The discriminator of the cWAGAN plays a role in discriminating the predicted accuracy of the generator during the training stage. Moreover, in the test or deployment stage, the discriminator is dropped. Choi and Cha [44] proposed an encoder–decoder architecture that was inspired by existing architectures such as DenseNet and DeepLabV3+ [45] to segment the cracks from concretes. This architecture can also be regarded as a modified FCN for the purpose of binary segmentation of concrete cracks.

From the above-mentioned examples, it can be seen that most of the recent applications for pixel-level crack segmentation implemented FCN or its modified versions (encoder–decoder architectures). It might be available to implement a similar FCN architecture to achieve the end-to-end fatigue crack semantic segmentation in steel bridges. The possible obstacle is that in most of the images of steel bridges (e.g., steel girders), there are hand-writings or other markers that were made during past visual inspections to illustrate the size and path of the fatigue cracks. However, in the cases of concrete or pavement crack detection, especially in the datasets of the published applications, the backgrounds of the cracks are relatively simpler than the cases of fatigue cracks in steel structures. In addition, the fatigue cracks in steel structures are smaller than the cracks in pavements and concretes and other types of defects. Moreover, in this study, the images taken for fatigue crack detection are several times larger than those reported in the literature. This is another challenge. Compared with the applications of other deep learning methods for crack detection reported in the literature, the major contribution and novelty of this study is to integrate the deep-learning-based semantic segmentation methods with conventional image processing operations to segment the tiny fatigue cracks in steel structures/bridges from the complicated backgrounds. Image cropping and data filtering will be used to reduce the imbalanced data ratio between the background and the fatigue cracks in images and improve the segmentation performance.

## 3. Methodology

**Figure 1:**
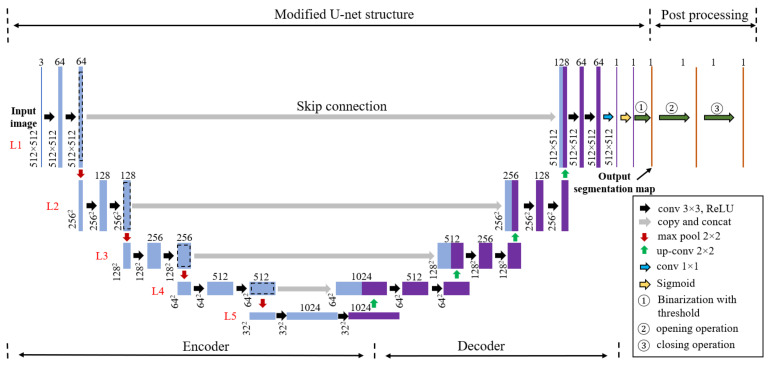
Overview of the proposed method for fatigue crack segmentation (modified from the original U-net [36], copyright Springer, 2015).

As stated in the last section, most current applications applied the FCN or the modified versions to achieve crack segmentation at pixel level. FCN and most of its modified versions were developed for the purposes of multi-class segmentation, and during the implementation in crack segmentation, the number of segmentation classes was reduced to two: one is the class of crack and the other is the class of background, which includes all the objects except the cracks in the images. U-net [36] was originally designed as an architecture for binary semantic segmentation and edge-like objects, which makes it much more straightforward for crack detection. Furthermore, U-net is suitable for the training cases when the size of the dataset is small. All the advantages mentioned above make U-net a good fit for the scope of this study. In this study, a deep neural network modified from U-net is implemented for the semantic segmentation of fatigue cracks in steel structures. Figure 1 shows an overview of the proposed method for fatigue crack segmentation. It consists of two major parts: modified U-net structure and post-processing.

### 3.1. The Modified U-Net Structure

The modified U-net structure includes an encoder part and a decoder part. In the original U-net [36], the input image is in grayscale with the size of 572 × 572 pixels (the unit, pixels, are omitted for simplicity hereafter). Because no padding is added in the convolution operation, after each convolution operation, the map size of each side is reduced by 2. This results in the size of the final output of the crack segmentation map being smaller than the input image. The size here is related to the resolution, and it refers to the height and width of the image/map. In this study, the input is a three-channel colorful image with the size of 512 × 512. Zero padding with the size of one is added during each convolution operation to make sure the size of the final output is the same as the input image. It should be noted that although the input image size here is 512 × 512, images with random sizes can also work. This is one of the advantages of the FCN-like structures.

As shown in Figure 1, the modified U-net structure contains an encoder part and a decoder part. In the first level (L1) of the encoder, the input image first goes through a set of convolution operations with sixty-four (64) 3 × 3 × 3 kernels, and then the 64 feature maps go through the rectifier linear activation function (ReLU, Rectified Linear Unit). This procedure is denoted as “conv 3 × 3, ReLU”. It should be noted that only for the first “conv 3 × 3, ReLU”, the kernel size for convolution is 3 × 3 × 3. For the others, it is 3 × 3. The first “conv 3 × 3, ReLU” produces a feature map group with a number of 64 and a resolution of 512 × 512. Repeating the same procedure produces a feature map group with the same number and size as in L1. Then, after a set of max pooling operations with 64 2 × 2 kernels, it produces the last feature map group in L1 consisting of sixty-four (64) feature maps, but the resolution of each feature map is reduced to 256 × 256. The stride of convolution operation is 1 and the stride of max-pooling operation is 2. This group of feature maps is regarded as the input of L2. Then, it goes through two sets of “conv 3 × 3, ReLU” operations with 128 3 × 3 convolution kernels and 128 2 × 2 max pool kernels to get a feature map group with the number of 128 and the resolution of 256 × 256. This procedure is similar to L1. In different levels of the encoder part (L1 to L5), the operations of “conv 3 × 3, ReLU” and “max pool 2 × 2” repeats until the bottom level: L5. With the conv-ReLU-pooling operations, the resolutions of feature maps decrease, but more global information is included in the feature maps.

The last feature map group of L5 (denoted by the purple rectangular) consists of 1025 32 × 32 feature maps. In a classical CNN for classification, there is usually a set of fully connected layers and softmax layer to produce a vector of class scores after this feature map group. With the scores, the classification can be predicted at the image level (global level). However, in semantic segmentation tasks, the goal is actually to achieve the pixel-level classification. FCN and its modified versions or U-net apply a series of upsampling and feature fusion operations to increase the resolutions of feature maps and obtain the final semantic segmentation map. In this study, a set of deconvolution operations with 512 2 × 2 kernels for the purpose of upsampling follows the last feature map group of L5 (1024 32 × 32). It produces a feature map group with a number of 512 and a resolution of 64 × 64. By a feature fusion operation, which copies the last feature map group in L4 of the encoder and concats with the newly upsampled feature map group via a skip connection, a new feature map group with a number of 1024 and a resolution of 64 × 64 is produced. It should be noted that instead of “concat”, element-wise adding is used in the original FCN to conduct feature fusion. After two “conv 3 × 3, ReLU” operations with 512 3 × 3 kernels, two feature map groups with a number of 512 and a resolution of 64 × 64 are obtained in L4 of the decoder. The same procedure performed from L5 to L4 of the decoder is repeated through L4 to L1 of the decoder. During the procedure, the number of the feature map in each group decreases but the resolution of the feature map increases. Finally, two feature map groups with the number of 64 and the resolution of 512 × 512 are obtained, which is the same as with the input image. The last feature map group follows a set of convolution operations with 64 1 × 1 kernels to produce a feature map with the size of 512 × 512. Then, after going through the sigmoid function and image binarization using the threshold, the output segmentation map is obtained.

### 3.2. Post Processing

Basically, for the semantic segmentation task, the modified U-net structure can achieve the preset goal. However, in the prediction results, there might be cases that in a segmentation map, there are multiple small fatigue crack fragments that should be connected in one or there are some tiny fragments that are just noise. The segmentation map of the fatigue cracks needs to be further processed to solve the problems abovementioned. As shown in Figure 1, a “post-processing” procedure is added after the modified U-net structure. The opening operation is performed to eliminate the noises, and the closing operation is performed on the output segmentation map to connect the small fatigue crack fragments. At the end, the final fatigue crack segmentation result is obtained. In the opening operation, the image first conducts the erosion and then the dilation operation. The erosion operation erodes the boundaries of the foreground object (here, it refers to fatigue crack) and makes the foreground object smaller. While the dilation operation enlarges the boundaries of the foreground object and makes the foreground object larger. The closing operation is just the opposite of the opening operation. In the closing operation, the image first conducts the dilation and then the erosion operation.

## 4. Dataset Generation

**Figure 2:**
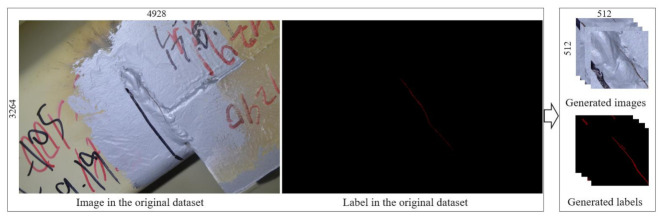
Overview of dataset generation.

The original dataset was granted by the organizing committee of the 1st International Project Competition for Structural Health Monitoring (IPC-SHM 2020) [46,47]. It includes 120 image–label pairs for training and testing purposes and additional 80 original images without the labeled part. Eighty of the 120 image-label pairs are selected as the training dataset, and the remaining 40 are selected as the test dataset. The resolutions of the original images are 4928 × 3264 pixels and 5152 × 3864 pixels, which are too large for direct training and testing. Training or testing directly using images with such large sizes would induce an out-of-memory error, even if a high-performance graphics processing unit (GPU) is equipped. Actually, in most of the deep-learning-based crack semantic segmentation applications in the literature, the size of the input image is much smaller than the examples in this study. For the sake of higher training and testing efficiency, in this study, it is necessary to preprocess the original 120 image–label pairs and generate a different appropriate dataset with a suitable resolution for training and test purposes. The resolution of the images in the new datasets would fit the size of the input image shown in Figure 1**.** The images in the original dataset are cropped into small image patches (generated images and generated labels) with the resolution of 512 × 512 as illustrated in Figure 2. As stated in [46], the original image dataset was obtained by the bridge inspectors with different camera and lens configurations. Then, the fatigue cracks were visually detected, and the fatigue crack regions were manually labeled in the images at pixel level. As shown in Figure 2, not only are the fatigue cracks shown in the images, but also the markers and annotations are presented to indicate the fatigue crack information. Since the fatigue cracks are very thin, sometimes it is hard to find them in Figure 2. Detailed image and label samples used in this study are found in [46].

The cropping goes from left to right and from top to bottom. Because the ratio between the original image and cropped image is not an integer, there are reminders on the right and bottom border after the cropping. During the training stage, the reminders are discarded. However, during the test stage, there are two test steps: (1) test on the cropped images, and (2) assemble the cropped image into the original large images. In the second step of the test, the remainders during image crop are directly fed into the network and used for prediction. The class imbalance of the background and the fatigue cracks is a problem in the original images. The class imbalance means that the ratio between the two classes is too large or too small. Filtering the cropped image data by excluding the images without fatigue cracks could improve the training efficiency and model accuracy. In the generated dataset, the cropped images without fatigue cracks are excluded. The original 80 large images for training generated 460 images with the resolution of 512 × 512 as the training dataset. The original 40 images for testing generated 244 images with the resolution of 512 × 512 as the test dataset. The final test will be conducted on the original test dataset by using image cropping and resembling.

## 5. Training Details

### 5.1. Training Setup

The presented fatigue crack segmentation procedure was performed on a workstation with the configuration of Intel(R) Xeon(R) E-2124G CPU @ 3.40 GHz, Nvidia Quadro P5000 (GPU with 16 Gb memory and 2560 CUDA cores), and 32 Gb RAM. The whole procedure was coded in Python programming language using PyTorch 1.60 deep learning framework.

### 5.2. Loss Function and Parameters Selection

To train the fatigue crack segmentation model (modified U-net structure), the Binary Cross Entropy loss function was used. Regarding the model optimization, the RMSprop optimizer was used, and the learning rate, weight decay, and momentum were set as 1 × 10^−5^, 1 × 10^−8^, and 0.9, respectively. The batch size was set as 4. The parameters of the modified U-net were initialized with the default methods in PyTorch (Kaiming uniform and normal). With these settings, the whole training duration was around 4.5 h.

## 6. Results Analysis and Discussion

### 6.1. Evaluation Indicators

The evaluation indicators used in this study are precision (*P*), recall (*R*), *F*1 score, and intersection over union (*IOU*). The cracks pixels in the labeled region are regarded as positive entities. The predicted results from the trained model generally have four cases: true positive (*TP*), false positive (*FP*), true negative (*TN*), and false negative (*FN*). The evaluation indicators abovementioned are defined as:

(1) $$P=\frac{TP}{TP+FP}$$

(2) $$R=\frac{TP}{TP+FN}$$

(3) $$F1=\frac{2\times P\times R}{P+R}$$

(4) $$IOU=\frac{Area\;of\;overlap\;of\;prediction\;and\;ground\;truth}{Area\;of\;union\;of\;prediction\;and\;ground\;truth}$$

### 6.2. Result Analysis

**Figure 3:**
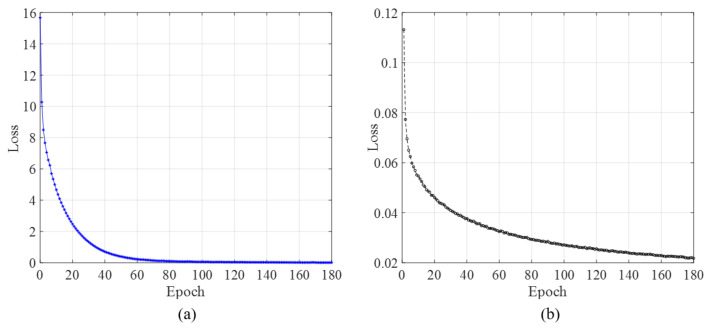
Loss curve of the semantic segmentation models in the training process: (**a**) U-net and (**b**) FCN.

To verify the developed method using U-net, in this study, FCN [30] was also implemented as the semantic segmentation model, and the Resnet34 [33] was selected as the backbone of FCN. Details of FCN can be found in [30]. The FCN was trained with the same dataset as presented in Section 4. Figure 3 shows the loss curves of U-net and FCN during the training process over epochs. From Figure 3a, it can be seen that in the first 20 epochs, the training loss drops dramatically, and after Epoch 20 (loss is 2.47), the loss decreases slowly. The loss at Epoch 90 is 0.076, which is already quite small. From Epoch 90, the loss of U-net gradually reduces to zero, and increasing the training epochs might give a trend of model overfitting. However, for the loss of FCN as shown in Figure 3b, after Epoch 90, the loss still gradually decreases, but the change is slow. For comparison purposes, the loss curves of the training of the two different models only keep 180 epochs.

The trained model weight parameters at selected epochs according to the loss curve in Figure 3 are used to predict the fatigue cracks in the generated test dataset using cropped images. Then, the predictions in the generated test dataset are assembled to obtain the results for the large images of the original dataset. The evaluation indicators are listed in Table 1. Here, mP, mR, mF1, and mIOU are the mean values of *P*, *R*, *F*1 score, and IOU, respectively.

**Figure 4:**
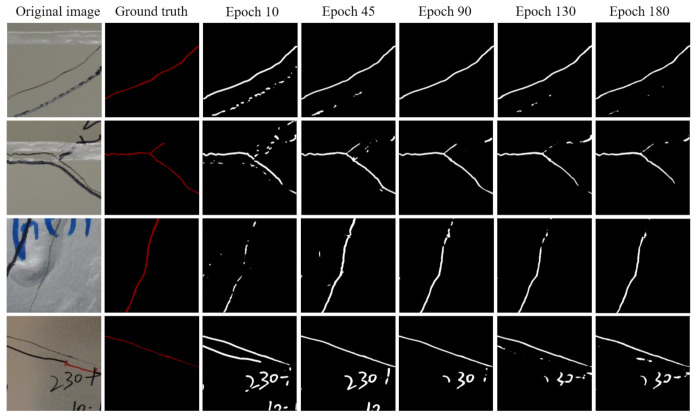
Predictions examples on the generated test dataset using the weights at different epochs: U-net.

For U-net, as shown in Table 1, before Epoch 90, the mean values of precision, recall, F1 score, and IOU go up gradually, but after Epoch 90, they begin to decrease slowly by small percentages. This is consistent with the observation in Figure 3a. Figure 4 lists the U-net prediction examples on the generated test dataset using the weights at different epochs. The listed examples represent most of the detection scenarios that include the obstacles that can be recognized as fatigue cracks such as marker curves, edges of weld lines, and handwritings. By comparing with the prediction results using the model weights obtained at different epochs and the ground truth, it can be seen that the prediction results at Epoch 90 give the best results. After Epoch 90, the noise level begins to increase. The noises here are mainly the false-positive small crack fragments, which are supposed to be the handwriting or markers. Combining Figure 3a, Figure 4, and Table 1, the weight parameters of the trained U-net model obtained at Epoch 90 can be used as the final prediction tasks, and this might be able to prevent model overfitting. Although the prediction results at every epoch is not shown here, Epoch 90 can be regarded as a good choice. It should be noted that there were some false-positive results such as handwriting “230” in the last row of the results in Figure 4. The possible reason might be that the features or textures of the handwriting in the original image are very similar to the fatigue cracks, and thus the handwriting was detected as fatigue cracks by mistake. This is one of the limitations, and the possible solution to improve the performance could be to enlarge the dataset with advanced data augmentation.

**Figure 5:**
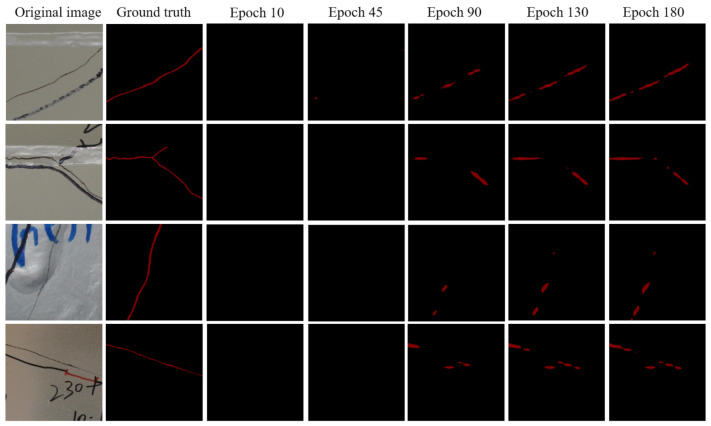
Predictions examples on the generated test dataset using the weights at different epochs: FCN.

For FCN, as shown in Table 1, before Epoch 90, the mean values of precision, recall, F1 score, and IOU increase very fast as the training epoch increases. After Epoch 90, the increase becomes slow. Figure 5 lists the FCN prediction examples on the generated test dataset using the weights at different epochs. By comparing with the prediction results using the model weights obtained at different epochs and the ground truth, it can be seen that the prediction results at Epoch 180 give the best in the first 180 epochs during the training of FCN. This is consistent with what is observed from the evaluation indicators as listed in Table 1. It should be noted that before Epoch 45, FCN can barely output fatigue crack prediction results, and in Figure 5, the result columns of Epoch 10 and Epoch 45 only show a black background. The result columns of Epochs 90, 135, and 180 show clearly identified fatigue crack fragments. Combining Figure 3b, Figure 5 and Table 1, the weight parameters of the trained FCN model obtained at Epoch 180 can be used as the final prediction tasks.

These observations in Figure 3, Figure 4 and Figure 5 and Table 1 indicate that FCN needs more training epochs to obtain a good prediction model. mIOU is the effective indictor for the evaluation of the performance of a trained semantic segmentation model. For FCN, it takes 180 training epochs to achieve an mIOU of 0.5774. However, for U-net, it only takes 90 training epochs to achieve an mIOU of 0.6479, which is about a 12% improvement compared to FCN. Another observation is that although FCN needs more training epochs to get a good prediction model, it does not identify the handwriting as fatigue cracks, as shown in the last row of Figure 5. This happens in the prediction of U-net as shown in the last row of Figure 4.

**Figure 6:**
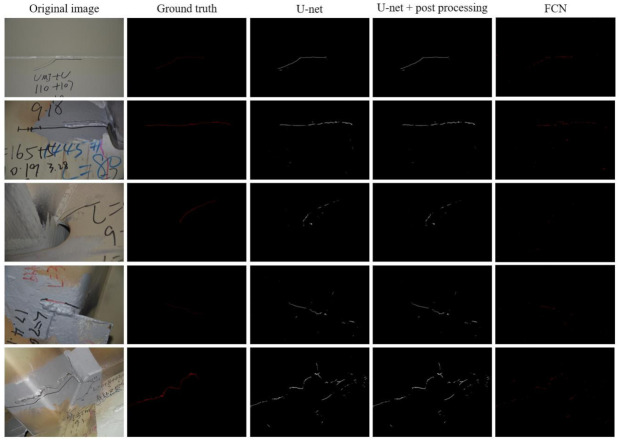
Prediction examples on the original test dataset with large images.

After the prediction on the test dataset using the trained U-net model, the post-image-processing, including opening and closing operations, is performed. The final fatigue crack semantic segmentation results are obtained by assembling the results in small images. Figure 6 shows several prediction examples by using U-net, U-net with post-processing, and FCN on the original test dataset with large images.

For U-net, most of the fatigue crack segmentation is good, except that in some of them, the marker curves, handwritings, and edges of weld lines are recognized as small fatigue crack fragments. Even using the post-image-processing, including opening and closing operations, the segmentation accuracy can be barely improved in terms of mIOU. The mIOU of the prediction just with the trained model is 0.6506, while after post-image-processing, the mIOU is also close to 0.6506. However, from the segmentation map in the fourth column of Figure 6, it is suggested that the noises were slightly eliminated. Some tiny fragments (negative–positive results) in the first and second rows of the prediction results from the trained model were removed, but there are still some other tiny fragments in the results after applying post-processing. In the last row, the prediction with the trained model gave some false-positive results, including some tiny fragments, and the tiny fragments were still there even after post-processing. An “L-shaped” segmented fatigue crack was regarded as the negative-positive prediction by using the trained model and after post-processing. In addition, the fatigue cracks in the second to fourth rows in Figure 6 should be in a continuous fashion, but the predictions from the trained model and after post-processing gave some disconnected fragments. The regions that should connect these disconnected fragments (true positive) are regarded as false negatives. It should be noted that the post-image-processing depends on the filter sizes and parameters, and further manual adjustment should be done to get better estimation results.

For FCN, as shown in the last column of Figure 6, it apparently provides a large amount of disconnected fragments of fatigue cracks in the listed example images. Compared to U-net and U-net with post-processing, FCN could not output better segmentation results with an acceptable accuracy at the current training stage. The comparative results indicate that the proposed method using U-net can achieve better segmentation performance with fewer training epochs. Moreover, the U-net structure implemented in this study is much simpler than FCN with ResNet 34 as the backbone, which is another advantage of U-net.

### 6.3. Discussions of the Considerations and Recommendations for Engineering Practices

**Figure 7:**
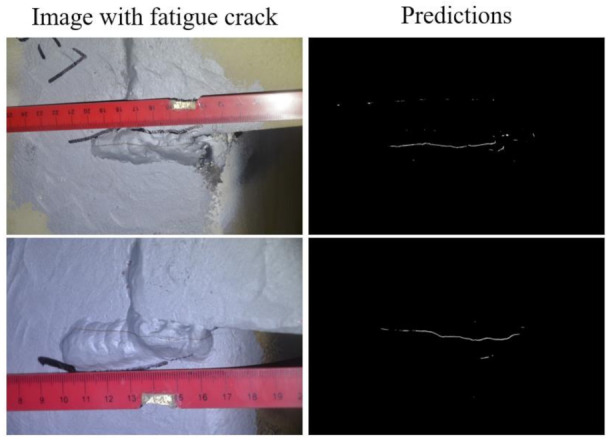
Fatigue crack predictions on the images with unknown objects in the training and testing dataset.

In the engineering practices of fatigue crack inspection, there are some considerations and recommendations when using the proposed methods or similar approach for automatic detection and segmentation:

(1) Large image problems. As presented above, in this study, the resolutions of the images in the dataset are quite large (4928 × 3264 pixels and 5152 × 3864 pixels), which makes it very difficult to train and test. The large images can be cropped into small images with a specific resolution for training and testing purposes. The final training can be obtained by assembling small images to the large images with the original resolution. The large images can also be downsampled to lower resolution, and then model training can be conducted. However, this option might lose the details of fatigue cracks, and it is very similar to the cases of using low-resolution cameras to collect images. Image downsampling is not selected and verified in this study.

(2) Model selection. There are many existing deep learning semantic segmentation models, but not all of them are suitable for fatigue crack segmentation. For engineering practices, especially from the civil infrastructure practitioners’ point of view, a simple end-to-end existing deep learning model for semantic segmentation is recommended by the authors.

(3) Model generality. The pretrained model for fatigue crack detection with the existing dataset might not always work. This means that it might be a lack of generality. For example, as shown in Figure 7, the ruler in the image is not included in the training dataset, and it takes a large portion in the images, even much larger than the fatigue cracks. The edges or shadows of the ruler have similar features with the fatigue cracks, and when using the pretrained model to predict the fatigue cracks in such images, the edges and shadows of the ruler might be recognized as cracks by mistake. Although the predictions of the trained model perform well and the noises are small in the listed examples, there is still the possibility that the model might need to be updated by retraining using the new dataset collected from new application scenarios.

(4) Estimation of geometric information of detected fatigue cracks. The developed method in this study is for pixel-level semantic segmentation of fatigue cracks, and currently, without camera calibration information, it could not give the geometric information of segmented fatigue cracks such as width and length. Camera calibration with assisted tools such as a marker board or a calibrated stereo camera set can be used to get the relationship between the image coordinate and the physical world coordinate and estimate the geometric information of fatigue cracks from segmentation results.

## 7. Conclusions

This study presents a pixel-level fatigue crack segmentation framework for large-scale images of steel structures using an encoder–decoder network. The encoder–decoder network is a modified version of an existing deep learning semantic segmentation model, i.e., U-net. By combining the trained deep learning model with post-image-processing operations, the proposed framework can obtain acceptable segmentation results of tiny fatigue crack in steel structures from large-scale images with complicated backgrounds. The main approaches, findings, and conclusions are as follows:

(1) To segment the fatigue cracks in the images with large resolutions such as 4928 × 3264 pixels or larger, image crop and assemble are applied during the training and testing stages. To prevent model overfitting, the training weight at a suitable epoch should be selected carefully for accurate and reliable fatigue crack prediction.

(2) Post-image processing such as opening and closing operations can eliminate the tiny noises, but the improvement in terms of mIOU is limited. The operations need manual adjustment. The mIOU of the semantic segmentation on a test dataset with 40 images is 0.6506 when using a presented model trained by 80 images.

(3) An FCN model with ResNet 34 was also trained with the same dataset, and the comparative results suggest that the proposed method using U-net could give better fatigue crack segmentation performance with fewer training epochs and a simpler model structure. The U-net gives a 12% improvement in mIOU with only 90 training epochs compared to FCN with 180 training epochs.

(4) The marker curves, edges of weld lines, and handwritings might be detected as small fatigue crack fragments by using the proposed method, and it is difficult to fully eliminate them with more training epochs or post-processing.

(5) The model trained in this study might give wrong predictions when the test image has a large difference from the training dataset (different surface texture, crack width, the human-made drawing, lighting conditions, etc.). A possible solution is to acquire more datasets in different application scenarios, implement data augmentation to expand the dataset, and retrain the model.

This paper also provides helpful considerations and recommendations for researchers and engineering practitioners in the field of civil infrastructure engineering. There are still limitations in the proposed framework. In the future, more deep learning model structures and dataset generation approaches will be investigated to eliminate the false-positive fatigue crack fragments and improve the generality and calibration information of a single camera or a stereo camera set, which will be utilized to estimate the geometric information of fatigue cracks.

## Figures and Tables

**Table 1:** Evaluation indicators on test dataset using the trained weight parameters at different epochs.

	Epoch	10	45	90	130	180
U-net	mP	0.3365	0.3498	0.4423	0.4577	0.4457
	mR	0.3712	0.5635	0.5209	0.5145	0.5313
	mF1	0.2958	0.3744	0.4295	0.4172	0.4279
	mIOU	0.5960	0.6248	0.6506	0.6430	0.6479
FCN	mP	0.1995	0.4163	0.5199	0.5194	0.5693
	mR	0.0032	0.0578	0.1226	0.1686	0.1942
	mF1	0.0032	0.1016	0.1985	0.2546	0.2896
	mIOU	0.4939	0.5190	0.5475	0.5654	0.5774

## Data Availability

The fatigue crack dataset used in this study was provided by IPC-SHM 2020. More information can be found in [46].
